# Impact of Epigenome-Wide Methylation and Breast Cancer Recurrence in Women Tested Negative for BRCA Genes: The Breast Methylation Risk (BREMERI) Study

**DOI:** 10.3390/cancers17193132

**Published:** 2025-09-26

**Authors:** Silvia Polidoro, Harriet Johansson, Giovanni Cugliari, Aliana Guerrieri-Gonzaga, Valentina Aristarco, Debora Macis, Mariarosaria Calvello, Monica Marabelli, Irene Feroce, Davide Serrano, Sara Cagnacci, Cristina Zanzottera, Francesca Fava, Federica Bellerba, Bernardo Bonanni, Sara Gandini

**Affiliations:** 1 Department of Translational Medicine, University of Piemonte Orientale, 28100 Novara, Italy; silvia.polidoro@uniupo.it; 2 Division of Cancer Prevention and Genetics, European Institute of Oncology, IEO, IRRCCS, 20141 Milan, Italyvalentina.aristarco@ieo.it (V.A.); debora.macis@ieo.it (D.M.); irene.feroce@ieo.it (I.F.); davide.serrano@ieo.it (D.S.);; 3 Department of Medical Sciences, University of Turin, 10124 Turin, Italy; 4 Department of Experimental Oncology, European Institute of Oncology, IEO, IRRCCS, 20141 Milan, Italy; federica.bellerba@ieo.it (F.B.); sara.gandini@ieo.it (S.G.)

**Keywords:** epigenetics, DNA methylation, glucose homeostasis, breast cancer

## Abstract

Background and Aim: DNA methylation may contribute to a worsening in breast cancer (BC). Methods: We conducted a matched case–control study to investigate the contribution of DNA methylation (DNAm) in breast cancer recurrence risk. Genome-wide DNAm profiles were generated from peripheral white blood cells (WBC) collected post-surgery from women with primary breast cancer *BRCA* wild-type, using Illumina Infinium HumanMethylationEPIC array. Cases had to experience recurrence of breast cancer or death and were matched to controls (subjects without recurrence, ratio 1:2) by age at diagnosis (+/− 5 years) and follow-up duration. Results: We identified three differentially methylated regions between the groups. Cases showed two hypomethylated regions, one upstream of the *vtRNA2–1* gene (estimate −0.30, *p*-value < 0.005), and one in the 5′ UTR region of the *FGFR2* gene (estimate −0.34, *p*-value < 0.028), whereas one, upstream of the *RUFY1* gene (estimate 0.32, *p*-value < 0.015), was hypermethylated. Additionally, we identified two methylation signals, recognised as predictors of biochemical traits. The chemokine ligand 21 (unadjusted *p*-value < 0.03) and insulin receptor expression (unadjusted *p*-value < 0.04) were higher in cases than in controls. Conclusions: Our exploratory study suggests that specific DNA methylation patterns in WBCs, particularly in genes related to cellular proliferation, invasion, and glucose homeostasis, may be associated with the risk of breast cancer recurrence in *BRCA* wild-type women. If validated in larger cohorts, these circulating signatures may serve as blood-based biomarkers to improve risk stratification and guide tailored treatment strategies.

## Simple Summary

Breast cancer can sometimes return after initial treatment, but predicting who is most at risk remains difficult. Our matched case–control study examined DNA methylation, defined as chemical variations in the DNA that influence gene activity. We analysed blood samples from women with breast cancer who did not carry BRCA mutations, comparing those who later had recurrence with those who remained cancer-free. Three DNA regions showed different methylation patterns between the two groups. These regions contain three genes known to contribute to important processes such as cell growth, invasion, and how the body metabolises sugar. These findings suggest that DNA methylation patterns in blood could one day help identify women at higher risk of recurrence and guide more personalised follow-up and treatment strategies.

## 1. Introduction

Despite breast cancer having the highest incidence of all cancers in women worldwide, patients’ life expectancy is much higher compared to many other cancers. Breast cancer is a heterogeneous disease resulting from differences in several cancer-cell-intrinsic parameters, including genetic profile, the interplay between the genome, epigenome, and transcriptome, as well as proteome, migration and invasion capabilities, proliferation, stemness, and intrinsic cell plasticity [1]. Among breast cancer patients meeting the criteria for genetic testing of hereditary breast and ovarian cancer syndrome, approximately one-fourth are carriers of a pathogenic variant in *BRCA1* or *BRCA2* (*BRCA*) genes, whilst other less common susceptibility genes may also contribute to increased disease risk (*TP53*, *PTEN*, *STK11*, *PALB2*, *ATM*, and *CHEK2*) [2]. However, many patients with suspected hereditary breast cancer remain without a clear etiopathogenetic origin of the disease.

DNA methylation is recognised as a stable, heritable mark that can affect genome function and influence gene expression. Indeed, it is also shaped by environmental conditions. White blood cell (WBC) DNA methylation motifs have shown promise as biomarkers for assessing breast cancer susceptibility in multiple-case breast cancer families without known germline mutations [3].

Several genome-wide studies have found evidence of an association between global DNA methylation levels and an increased risk of breast cancer, detectable years before a clinical diagnosis [4,5,6]. The epigenetic machinery has been shown to play an essential role in carcinogenesis and progression through aberrant DNA methylation and histone modifications at the tissue level, exhibiting significant heterogeneity across different cancer types in this rapidly expanding field of research [7].

In breast cancer patients, specific methylation patterns could affect the risk of recurrence. In a genome-wide search for DNA methylation markers using available archival tumour samples from node-negative triple-negative breast cancer (TNBC), elevated levels of DNA methylation were associated with shorter recurrence-free intervals in triple-negative breast cancer [8], pointing to a pivotal role of methylation changes in the transition from primary breast cancer to metastatic disease by regulating key genes implicated in tumour suppression and oncogenic activity. Notably, different breast cancer subtypes exhibit different metabolic phenotypes, and altered metabolisms of glucose, lipids, or amino acids [9]. It is known that cancer cells can change their energetic strategy to support rapid growth and metastatic potential [10] and this mechanism can be fuelled by peripheral or constitutional factors.

Although epigenetic studies in cancer have focused on the effect of gene methylation mainly on breast tissue expression and metastasis [11,12], growing evidence exists on the feasibility of conducting studies on white blood cells [3,13]. Interesting results emerged from a study conducted in China [14] applying a multiplex blood-based assay targeting DNA methylation in peripheral blood mononuclear cells. Based on four methylation markers, they were able to distinguish early-stage breast cancer patients from age-matched normal females. The sensitivity of the test was 93.2%, the specificity 90.4%, with an area under the curve of 0.940.

Variations in blood DNA methylation result from a combination of internal factors, such as genetics, age, hormones, as well as external factors, including environmental toxins, smoking, stress, and lifestyle. Although more complex and indirect as a source of risk biomarkers, blood sampling, compared to tissue biopsies, offers a minimally invasive and straightforward approach for methylation analysis, suitable for clinical monitoring.

Hence, in consideration of recent evidence for an association of white blood cell methylation signatures with breast cancer risk and the hypothesis that they may have a role even in breast cancer recurrence, within a cohort of multiple-case, *BRCA*-negative breast cancer patients followed at the European Institute of Oncology (IEO), we performed a genome-wide DNA profiling to investigate whether specific methylation patterns in WBCs are associated with breast cancer recurrence using the Illumina MethylationEPIC BeadChip microarray for whole-genome DNA methylation profiling [15].

## 2. Materials and Methods

### 2.1. Study Design and Patient Characteristics

The present work is a retrospective case–control study of women undergoing *BRCA1* and *BRCA2* germline testing for breast cancer diagnosis at IEO. After oncogenetic counselling at the Division of Cancer Prevention and Genetics during the years 2001–2015, patients who were negative for *BRCA* testing and had a diagnosis of primary breast cancer were considered. Only women with a blood draw after surgery were eligible. Cases had to have a recurrence (ipsi- or contralateral breast cancer event, second breast tumour, metastasis) or death at least six months after surgery. No previous diagnosis of cancer was admitted. Cases were matched to controls (subjects without recurrence; ratio 1:2) by age at diagnosis (+/− 5 years) and follow-up duration. The IEO Institutional Review Board approved the study (IEO 1058), and participants signed an informed consent for research purposes.

### 2.2. DNA Methylation Assessment 

Whole EDTA-treated blood samples were drawn and stored at −80 °C until assayed. Genomic DNA was extracted from whole blood specimens using a QIAamp DNA blood kit (Qiagen, Valencia, CA, USA) according to the manufacturer’s instructions using the automated platform “QIAcube” (Qiagen, Valencia, CA, USA) and quantified using a NanoDrop spectrophotometer (Thermo Scientific, Wilmington, DE, USA).

Samples were shipped to the Italian Institute for Genomic Medicine laboratory, where DNA samples underwent preliminary quality control before bisulphite-converting 500 ng of each sample using the EZ DNA Methylation Lightning Kit (Zymo Research, Irvine, CA, USA), following the manufacturer’s instructions.

Bisulphite-converted DNA samples were randomly arranged on the Infinium HumanMethylationEPIC BeadChip (Illumina, San Diego, CA, USA) and processed following the manufacturer’s instructions.

After amplification, hybridisation, staining, and washing, the Beadchips (referred to as the 850k chip) were scanned with the Illumina iScan SQ instrument to acquire raw image intensities, which were saved as IDATs for further processing. To mitigate the batch effect and optimise the space on the arrays, each case and one of its matched controls were analysed on the same chip, while the second control was randomly allocated to a different chip.

#### 2.2.1. DNA Methylation Data Pre-Processing

Raw DNA methylation data generated from Illumina Infinium HumanMethylation EPIC arrays were imported and preprocessed using the ChAMP Bioconductor package (v 2.8.3) [16].

The average methylation value at each CpG locus, i.e., average “beta (β) value” ranging from 0 to 1, was computed as the ratio of the intensity of the methylated signal over the total signal (unmethylated + methylated).

To reduce artefacts and genetic confounding, initial quality control filtered probes with detection *p*-values > 0.01, fewer than 3 Beads in 5% of samples, mapping to multiple locations or on sex chromosomes, with known SNPs at CpG sites or single-base extensions; 742,521 CpGs passed QC.

Normalisation of probe intensities was performed using the Beta MIxture Quantile dilation (BMIQ) method to correct for probe design bias (Type I and Type II). Batch effects due to technical variability were identified and corrected using ComBat from the SVA package (v 3.56.0), using empirical Bayes frameworks while preserving the biological variation of interest.

After normalisation and batch correction, beta values were converted to M-values by logit transformation for improved statistical handling in downstream differential methylation analyses. The final preprocessed dataset was subjected to further analyses, including differential methylation position (DMP) and region (DMR) identification using ChAMP’s analytical workflows.

To estimate the proportions of major white blood cell (WBC) subtypes in our samples and adjust the statistical analysis for cellular heterogeneity among samples, we applied the Houseman reference-based deconvolution method. This approach utilises a reference matrix of methylation signatures from purified leukocyte subpopulations, modelling bulk methylation profiles as linear combinations of these reference signatures [17].

#### 2.2.2. Differentially Methylated Analysis

The statistical analysis was structured to identify and interpret patterns of differential DNA methylation in cases vs. controls, using both site-specific and region-based approaches.

(i) Differentially Methylated Probes (DMPs):

Differential methylation at individual CpG sites was assessed using the champ.DMP functions within the ChAMP Bioconductor package (v 2.8.3). A linear modelling framework implemented via the limma package (v 3.64.3) was then applied probe-wise to test for methylation differences between cases and controls. 

Empirical Bayes moderation of standard errors was applied to enhance the reliability of variance estimates, especially in small sample sizes like ours.

(ii) Differentially Methylated Regions (DMRs):

DMRs are defined as contiguous genomic regions containing multiple CpG sites that exhibit coordinated changes in methylation levels between comparison groups. 

They were identified with the Bumphunter algorithm, available through the champ.DMR function in the ChAMP package (v 2.8.3). This method detects DMRs by clustering nearby CpG probes based on their genomic proximity. For each cluster, Bumphunter applies a permutation-based test to determine if regional methylation levels differ significantly between cases and controls. Since it does not depend on initial differential methylation findings at individual CpG sites, this approach is ideal for exploratory analyses.

Differentially methylated regions (DMRs) were visualised using the DMR.plot function from the ChAMP Bioconductor package (v 2.8.3) with default parameters. In these plots, the *X*-axis corresponds to the genomic coordinates of CpG probes within each DMR, and the *Y*-axis represents DNA methylation β values ranging from 0 (fully unmethylated) to 1 (fully methylated). For each comparison group, individual probe-level β values (“C” and “T” tracks) are shown alongside their group-wise means (“C mean” and “T mean”) and LOESS-smoothed curves (“C loess” and “T loess”). The averaging and smoothing steps are applied for visualisation only to highlight local correlation and regional methylation trends; all statistical inference of DMRs was performed at the probe level without smoothing.

(iii) Sensitivity Analyses on DMRs:

For the three identified DMRs, we investigated possible deviation from multimodality of the distribution of methylation β values at all CpGs within each region. We used Hartigan’s dip test, as implemented in the diptest R package (v 0.77-2) [18]. CpGs showing evidence of multimodality were further categorised using the mclust R package (v 6.1.1) [19]. Briefly, mclust applies a model-based clustering framework fitting Gaussian mixture models with varying numbers of components, selecting the optimal solution based on the Bayesian Information Criterion (BIC). Finally, logistic regression analyses were repeated for each CpG using the derived categorical variables, adjusting for age, array position, and estimated WBC, for consistency with the main analysis, to evaluate robustness of the results. All statistical analyses were conducted using the open-source software R (v 4.5.1).

(iv) Differentially Methylated Blocks (DMBs):

DMBs are large genomic regions composed of clusters of CpGs, typically spanning tens to thousands of kilobases. In ChAMP, DMB analysis first groups probes within open sea regions into larger contiguous blocks by averaging methylation values of neighbouring CpGs. The Bumphunter algorithm is then applied to these blocks to identify regions showing significant methylation differences between groups using permutation testing. Compared to DMR analysis, which targets smaller, densely packed CpG clusters, DMB detection focuses on broader methylation changes, improving sensitivity to large-scale epigenetic alterations. 

To account for technical variability and biological confounders, all models incorporated multiple covariates. In particular, adjustments were made for age at blood draw, chip position to control for batch effects, and for white blood cell (WBC) composition, which reflects inter-individual variability in cellular makeup—a known source of epigenetic heterogeneity in blood-based analyses.

Resulting *p*-values were corrected for multiple testing using the Benjamini–Hochberg false discovery rate method. Probes, regions, or blocks with adjusted *p*-values < 0.05 were considered statistically significant.

#### 2.2.3. Deriving Methylation-Based Surrogates

Methylation profile scores (MPS), also known as surrogate estimators or ‘predictors’ of human traits, have been calculated using the ‘MethylDetectR’ online tool (https://shiny.igmm.ed.ac.uk/MethylDetectR, accessed on 17 July 2025). 

These MPS have been calculated using machine learning methods to link DNAm data in a specific tissue (typically blood) to traits of interest, such as smoking status, epigenetic age, and concentrations of various biomarkers in plasma. These models identify CpG sites strongly correlating with the trait, assigning weights to each site’s predictive value. In our study, DNAm levels at key CpG sites were used to estimate trait values or scores for 117 human traits [18]. Linear regression was used to assess the associations between the MPS and breast cancer progression. Age at blood draw, chip position, and percentage of WBCs were controlled as potential confounders. For all tests, the significance level was set at *p* < 0.05, and *p*-values adjusted for FDR are presented.

All statistical analyses were conducted using the open-source software R (v 4.4.1).

## 3. Results

**Figure 1:**
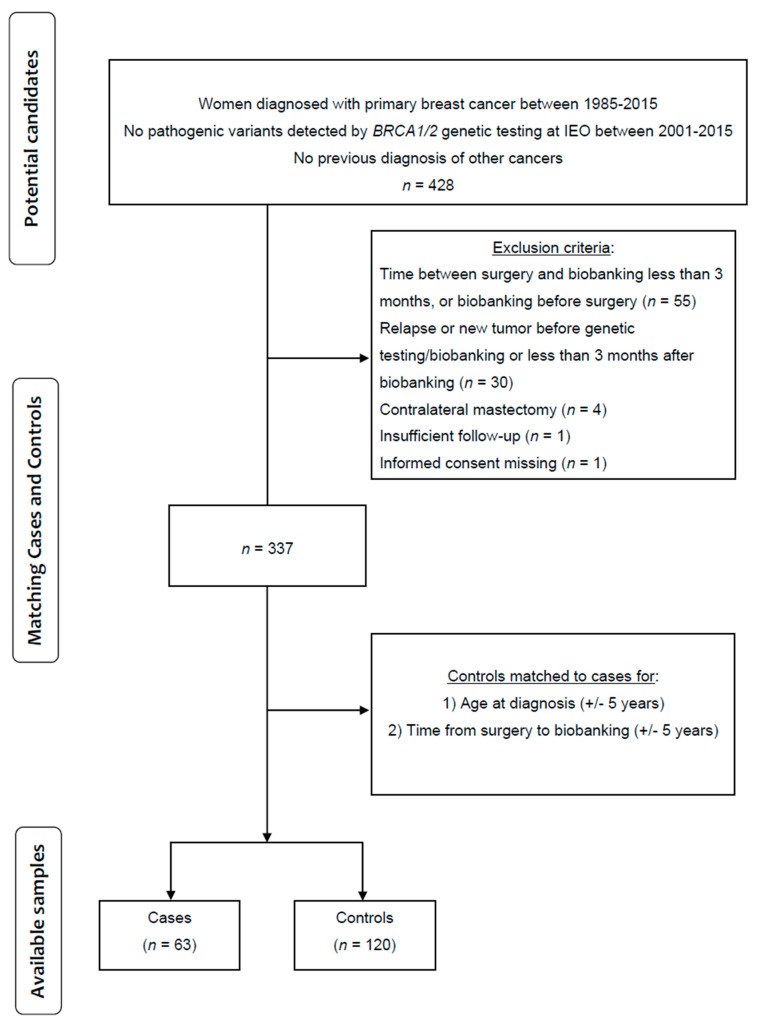
Patient Flow Diagram.

Overall, 428 candidate patients were considered. Figure 1 illustrates the additional exclusion criteria, reducing the cohort to 337 women. The cases were matched to controls (women without any breast cancer event or progression) by age at diagnosis (+/− 5 years), time from surgery to biobanking (+/− 5 years), and follow-up time. Eventually, 63 cases were matched to 120 controls. Cases were matched to 2 controls, except for six cases for whom only a single control was available.

In Table 1A, we present the characteristics of the study population. There was no statistically significant difference (*p* = 0.43) between the median age at genetic testing, which was 42 years with an interquartile range (IQ) of 37–49 years in cases (*n* = 63) and 45 years with an IQ of 38–50 years in the control group (*n* = 120). Likewise, we did not observe any statistical difference in BMI at genetic testing between groups (*p* = 0.69). Nonetheless, comparing BMI reported at 18 years of age, we observed a significant difference, showing a higher BMI at 18 years (*p* = 0.04) in cases (20, IQ 18–21) compared to controls (19, IQ 18–20). We also describe data on the use of oral contraceptives, pregnancies, menopausal status, smoking, and family history.

### Epigenome-Wide Differentially Methylated Probes

After post hoc correction for FDR (adjusted *p*-value), no statistically significantly differentially methylated single CpG probes were recorded between groups; nonetheless, the first 100 single CpGs ordered by nominal *p*-value were extracted to look for trends and were assessed for biological relevance without finding any statistical significance related to breast cancer recurrence (Table S1, in the Supplementary Materials).

The clustering analysis revealed three differentially methylated regions (*DMRs*) associated with recurrence. *DMR1* showed overall hypomethylation (estimate = −0.30 compared to the reference group, adjusted *p* < 0.005), *DMR2* was hypermethylated (estimate = 0.32 compared to the reference group, adjusted *p* < 0.015), and *DMR3* was hypomethylated (estimate = −0.34 compared to the reference group, adjusted *p* < 0.028).

Table 2 lists each CpG probe mapping within the three DMRs. *DMR1* on chromosome 5 included 13 CpGs in the upstream area of the transcriptional start site of the *vtRNA2–1* gene, as well as a few intergenic regions. On the same chromosome, *DMR2* includes nine CpGs mapping in the upstream area of the transcriptional start site of the *RUFY1* gene, one in the 1st exon, and one in the 5′UTR region of the same gene. On chromosome 10, the *DMR3* included seven CpGs, covering sequences in the 5′UTR region of the *FGFR2* gene.

**Figure 2:**
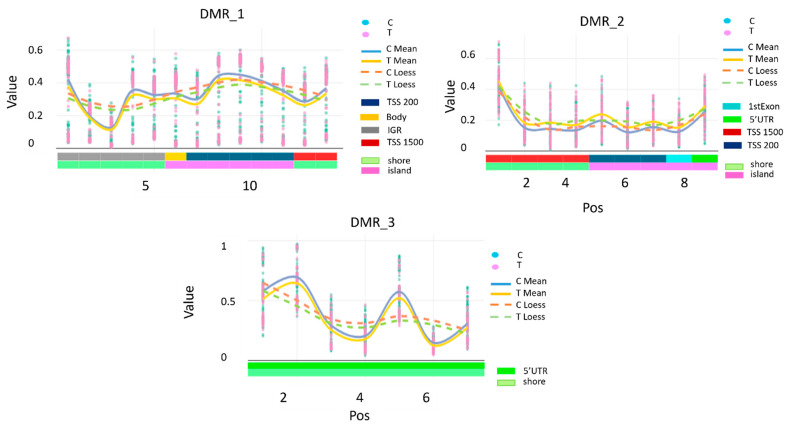
Difference in DNA Methylation levels of the three DMRs between groups. Each panel in the figure shows the methylation signal intensities of the CpGs within each DMR for cases vs. controls. “C” track: individual values of methylated signal intensities (corresponding to cytosine at the CpG site); “T” track: individual values of unmethylated signal intensities (corresponding to thymine signal when the CpG is not methylated). “C mean” and “T mean” tracks: average values of methylated and unmethylated intensities across probes within each group, plotted to reduce probe-level noise. “C loess” and “T loess” tracks: LOESS-smoothed curves of methylated and unmethylated intensities, applied to highlight regional trends in DNA methylation that reflect the local correlation between neighbouring CpGs.

Figure 2 shows the DNA methylation signal intensities of each CpG position across the three significant DMRs identified by ChAMP for cases vs. controls, highlighting the functional characteristics.

Given that CpGs showed evidence of multimodality (Figure S1, in the Supplementary Material), a sensitivity analysis categorising *DMR1* and *DMR3* was conducted to assess the robustness of the results. Logistic regression analyses, adjusting for age, array position, and estimated WBC, showed consistent results with the main analysis with ORs lower than one, even if the associations did not reach statistical significance, due to loss of statistical power (Table S2 in the Supplementary Material). The multimodal distribution of DNAm in *DMR1* and *DMR3* suggested a potential influence from methylation quantitative trait loci (meQTLs). To investigate this, we searched for nearby DNA sequence variants associated with the CpGs in these regions.

We consulted the EPIGEN MeQTL Database (https://epicmeqtl.kcl.ac.uk, accessed on 17 September 2025), which includes a comprehensive meQTL analysis of blood samples from 2358 individuals. According to this database, both *DMR1* (6 out of 13 CpGs in the region) and *DMR3* (all 7 CpGs in the region) contain genetic variants that are statistically associated with DNAm levels, with a false discovery rate (FDR) of less than 0.05 (Table S3, in the Supplementary Material).

Differentially methylated blocks (DMBs) analysis did not show any statistically significant differentially methylated blocks between groups after post hoc correction. Nevertheless, we detected a block (adjusted *p*-value = 0.05) that covers 327 CpG probes mapping within the genomic region of cadherin-4 (CDH4), a member of the cadherin family that encodes Ca^2+^ -dependent cell–cell adhesion glycoproteins, which could be of biological interest.

The surrogates’ results for the 117 MPS traits did not show statistically significant differences between cases and controls after adjusting for chip number, position, WBC percentage, and age, and after controlling for FDR. Nonetheless, we identified two traits with nominal significance: CCL21, a chemokine ligand (*p*-value = 0.028), and the insulin receptor (*p*-value = 0.041), both with a positive estimated coefficient (Table S4, in the Supplementary Material).

## 4. Discussion

In our study, although no association was found between isolated differentially methylated CpGs and breast cancer recurrence in *BRCA*-negative patients, the analysis of DMRs revealed three genomic segments that were significantly differentially methylated between cases and controls.

Two of these regions (*DMR1* and *DMR3*) showed a multimodal distribution of DNA methylation. This pattern could reflect the influence of methylation quantitative trait loci (meQTLs), although our data do not allow us to test this directly. Future studies are required to clarify this, but it is of interest that both regions coincide with meQTLs reported in the EPIGEN MeQTL database.

To further assess the robustness of our findings, we performed a sensitivity analysis by categorising DMR1 and DMR3. Logistic regression results suggested a pattern consistent with the primary analysis, with odds ratios below one. However, the associations did not reach statistical significance, likely reflecting the reduced statistical power of this additional analysis.

Interestingly, these segments span genes involved in cell signalling, mitogenesis, differentiation, and glucose metabolism regulation, all of which play a crucial role in modulating the risk of progression and metastasis.

The VAULT 2 locus, located on chromosome 5q31, encodes vtRNA2–1, also known as non-coding 886 (nc886), because it transcribes a non-coding RNA. This gene was recently investigated in a review [19] that highlights its distinctive characteristics, namely being the only human gene known to display maternal polymorphic imprinting [20]. The vtRNA2–1 methylation pattern of an individual tends to remain stable across a lifespan [21] and similar in many somatic tissues [20,21]. Furthermore, its RNA expression is associated with its methylation status [22]. In individuals with a non-methylated vtRNA2–1, the levels of RNAs [23] were approximately two-fold compared to individuals with an imprinted locus [23]. These unique characteristics of the vtRNA locus make it particularly interesting as a blood methylation biomarker of disease.

The expression of vtRNA2–1 has been described as upregulated in many cancers, including breast and prostate cancer [19]. This overexpression correlates with progression and poor prognosis in the latter [24]. vtRNA2–1 is known to bind and modulate the activity of target proteins like Protein Kinase R (PKR), 2’-5’-oligoadenylate synthetase 1, and Dicer [25] in complex regulatory mechanisms sensed through its methylation status. Moreover, the *vtRNA2–1* methylation status is associated with glucose and insulin levels during adolescence and glucose metabolism later in adulthood [22], linking methylation patterns with metabolic traits often associated with familial risk [3]. Notably, the *vtRNA2–1* has been suggested to have a role in post-transcriptional regulation of epithelial barrier integrity through the inhibition of the expression of tight junction and adherens junction proteins [26]. Specifically, elevated levels of *vtRNA2–1* were shown to reduce the levels of claudin 1, occludin, and E-cadherin, resulting in an impairment of the intestinal barrier. Impaired intestinal barriers can increase breast cancer prognosis by promoting chronic inflammation, facilitating bacterial translocation into the bloodstream, and influencing treatment response through gut microbiota dysbiosis, potentially increasing the risk of poor breast cancer prognosis [27]. In our study of women with breast cancer, the DMR of *vtRNA2–1* was more frequently hypomethylated in cases compared to controls. Thus, we may speculate that overexpression increases the risk of recurrence. Based on the consistency between studies, this hypothesis is reasonable [28]. Nonetheless, we acknowledge that our findings should be interpreted with caution, as some studies, mainly from tissue, found that hypermethylation in the promoter region of *vtRNA2–1* was associated with worse prognosis [19,29,30], because we were unable to study vtRNA2–1 expression in our cohort.

Along the same chromosomal region 5q31, another DMR segment covering the upstream area of the transcriptional start site of the *RUFY1* gene was hypermethylated in cases compared to controls in our cohort. To our knowledge, very few pieces of evidence have been published on DNA methylation status in peripheral blood mononuclear cells (PBMCs) describing DMRs of *RUFY1* and associations with disease. One study [31] found a DMR located on the *RUFY1* gene, showing an association between increased DNA methylation in the *RUFY1* promoter region and IgE-sensitised children, compared to non-sensitised children, consistently detected also in maternal PBMCs, and cord blood. An explanation linking the hypermethylation of the *RUFY1* gene to its downregulation in specific tissues and respective associations with disease is still to be supported. 

RUFY adaptor proteins, including RUFY1, are involved in complex biochemical crosstalk between the cytoskeleton and endosome dynamics, including the recycling of cytoplasmic materials [32]. Char and Pierre’s review [32] concluded that the dysregulated expression of RUFY proteins may severely affect cell differentiation and polarisation, contributing to the development of several cancers and neurodegenerative diseases. *RUFY1* has been identified as a downstream effector of Etk protein kinase, and its principal function is to bind PIP3-containing phospholipid vesicles that participate in early endosomal membrane trafficking and P13K signalling [33], mediating cell proliferation, colony formation, apoptosis, migration, and invasion. Still, evidence of a link between the hypermethylated region of RUFY1 in WBC and reduced cell expression in any tissue type is warranted before we can speculate on any specific mechanisms.

The third genome segment contains the *FGFR2* gene, which is frequently altered in tumours [34]. In our cohort of cases, we found that *FGFR2* was hypomethylated in the 5’UTR island region compared to the controls. This gene plays crucial roles in development and tissue repair [34], particularly in bone and blood vessels. Aleyasin and colleagues identified the *FGFR2* gene as a blood-based epigenetic biomarker in gastric cancer [13]. Consistent with our results, the *FGFR2* gene exhibited considerable hypomethylation in its CpG site, suggesting an epigenetic enhancement of FGFR2 expression [35]. *FGFR2* has been reported to be selectively upregulated to maintain the survival of dormant residual tumour cells [35], as well as to promote ERK1/2 signalling and ultimately recurrence. 

CDH4, which encodes R-cadherin, was the only gene mapping in a block of 327 CpGs that we found differently methylated, mainly hypomethylated, in our case–cohort compared to controls, which could have some interesting biological implications. The protein is a classical cell surface glycoprotein that plays a crucial role in cell-to-cell adhesion [36]. The protein is a calcium-dependent cell–cell adhesion glycoprotein expressed in the mammary epithelial cells of the ducts and lobules [37] and involved in epithelial-to-mesenchymal transition (EMT), a crucial step towards metastasis formation. CDH4 messenger RNA levels are described to be overexpressed in breast cancer cells compared to normal cells and correlate with poor prognosis in distant metastasis-free survival [38]. Nonetheless, CDH4 was described to be repressed during EMT in breast cancer cells [37] and downregulated in breast tumour tissue [39]. However, although our finding is suggestive of further investigations, we cannot directly translate the trend of hypomethylation of the block towards an increased transcriptional activity of CDH4 in tissue. Additionally, we did not find any evidence in the literature of a link between hypomethylated regions of the *CDH4* gene and reduced cell expression in any tissue type.

To better characterise our hypothesis that breast cancer recurrence is associated with altered methylation patterns, we used the methylation data to calculate a panel of surrogates for relevant metabolic traits. Regrettably, none showed a statistically significant difference after correction for multiple comparisons; nevertheless, two surrogates showed nominal significance and were estimated to be increased in cases compared to controls. 

The first is Chemokine (C–C motif) ligand 21 (CCL21), a chemokine ligand that seems to play a role in breast cancer progression, mainly through its interaction with the CCR7 receptor, crucial in various processes, such as tumour cell migration, lymphangiogenesis, and immune modulation, collectively contributing to breast cancer metastasis and progression [40,41]. The second protein estimated via DNA methylation surrogates showed a significant nominal increase in the Insulin receptor (IR). It is well known that the levels of IR are significantly higher in breast cancer tissues compared with healthy ones [42]. In the last decade, the role of the receptor in breast cancer, its involvement in tumour progression, and its potential as a therapeutic target, particularly in combination with the IGF1 receptor (IGF1R) [43] have gained increasing attention.

Although *CCL21*, *CDH4*, and *IR* lost statistically significant associations after adjustments in our dataset, given the small sample size of our study, their established roles in immune cell recruitment, cell adhesion, and metabolic/growth signalling, respectively, provide a supportive framework for the central findings. For example, *CCL21*’s involvement in recruiting immunosuppressive immune cells could synergise with *VTRNA2–1*-mediated barrier disruption to create a microenvironment permissive to tumour progression. Similarly, *CDH4*’s influence on cell adhesion and migration aligns with the trafficking functions of *RUFY1*, while *IR*’s regulation of growth factor signalling may intersect with *FGFR2*-driven pathways. In summary, our results highlight *RUFY1*, *FGFR2*, and *VTRNA2–1* as central components in a network that integrates intracellular trafficking, growth factor signalling, glucose metabolism, and epithelial barrier regulation to drive tumour progression (Table 3).

A limitation of our retrospective study is the lack of blood samples collected before breast cancer diagnosis, which prevents us from determining whether the observed methylation patterns preceded or followed surgery of the primary tumour. Additionally, we anticipated reaching a sample size of 70 cases and 140 controls. However, after applying all necessary exclusion criteria and matching, we ultimately included 63 cases and 120 controls. BRCA wild-type breast cancer is a biologically heterogeneous group, expressing different metabolic phenotypes [9]. This heterogeneity may have contributed to the lack of significant results in the probe-by-probe analysis and the reduced likelihood of detecting strong, consistent DMRs across patients. To improve our knowledge of how altered methylation influences breast cancer risk or progression, further large prospective studies are needed, focusing on the role of peripheral WBC DNA methylation patterns in unaffected women to establish the role of differently methylated regions as breast cancer risk biomarkers and collecting breast cancer tissue at diagnosis. A longitudinal follow-up of breast cancer cases would enable researchers to study the prognostic value of these biomarkers. Despite these limitations, our exploratory study revealed consistency among results linking DMRs with metabolic traits in a high-risk cohort of breast cancer patients that could benefit from tailored treatment approaches based on our findings.

## 5. Conclusions

Although our results are of an explorative nature and need further investigation in larger prospective cohorts, they suggest that specific epigenetic patterns in white blood cells may signal a higher risk of cancer recurrence. According to our findings, altered methylation regions of genes involved in critical cellular processes, such as cellular proliferation, tissue development and repair, and cancer invasiveness, appeared to confer a greater risk of recurrence in women diagnosed with breast cancer without known pathogenic variants in the *BRCA1* and *BRCA2* genes. If confirmed, these methylation patterns could serve as biomarkers in patients without detectable mutations. 

## Figures and Tables

**Table 1:** (**A**) Patients’ characteristics. Tumour characteristics are described in (**B**), including histotype, molecular subtype, lymph node stage, and tumour stage. (**B**) Tumour characteristics.

**(A)**					
		**Cases**	**Controls**	**Total**	***p*-Values**
Total		63	120	183	
Age at the test	Median (Q1, Q3)	42 (37, 49)	45 (38, 50)	183	0.43
BMI	Median (Q1, Q3)	22.1 (20.2, 24)	22.1 (20.2, 24)	182	0.69
BMI at 18 years old	Median (Q1, Q3)	20 (18, 21)	19 (18, 20)	177	0.04
Time from surgery to blood draw (months)	Median (Q1, Q3)	12.5 (8.2, 29.2)	21.5 (9.4, 52.7)	183	0.085
Oral contraceptive	No	24 (39.34)	37 (60.66)	61	0.32
	Yes	36 (31.3)	79 (68.7)	115	
	Missing	7			
Pregnancies	No	15 (29.41)	36 (70.59)	51	0.39
	Yes	48 (36.36)	84 (63.64)	132	
*n*. of pregnancies	No	15 (29.41)	36 (70.59)	51	0.64
	One	17 (34.69)	32 (65.31)	49	
	More than one	31 (37.35)	52 (62.65)	83	
menopausal status	Pre	48 (33.8)	94 (66.2)	142	0.65
	Peri	0 (0)	1 (100)	1	
	Post	14 (38.89)	22 (61.11)	36	
	Missing	40			
Smoking	Former	15 (31.91)	32 (68.09)	47	0.68
	No	43 (36.75)	74 (63.25)	117	
	Yes	5 (27.78)	13 (72.22)	18	
	Missing	1			
Family history	No	0 (0)	2 (100)	2	0.26
	Breast only	59 (33.91)	115 (66.09)	174	
	Breast and ovary	4 (57.14)	3 (42.86)	7	
Family history—First degree breast	0	21 (43.75)	27 (56.25)	48	0.19
	1	34 (31.19)	75 (68.81)	109	
	2	7 (28)	18 (72)	25	
	3	1 (100)	0 (0)	1	
Family history—First degree ovary	0	61 (34.08)	118 (65.92)	179	0.61
	1	2 (50)	2 (50)	4	
Family history—Second degree breast	0	20 (28.99)	49 (71.01)	69	0.39
	1	32 (35.56)	58 (64.44)	90	
	2	9 (42.86)	12 (57.14)	21	
	3	2 (66.67)	1 (33.33)	3	
Family history—Second degree ovary	0	61 (34.08)	118 (65.92)	179	0.61
	1	2 (50)	2 (50)	4	
(**B**)					
		**Cases**	**Controls**	**Total**	***p*** **-Values**
Total		63	120	183	
Histotype	In situ	9 (40.91)	13 (59.09)	22	0.85
	Ductal	48 (34.29)	92 (65.71)	140	
	Lobular	4 (30.77)	9 (69.23)	13	
	Other	2 (25.00)	6 (75.00)	8	
Molecular subtype	Luminal A/B	40 (35.71)	72 (64.29)	112	0.69
	Luminal B Her2+	7 (31.82)	15 (68.18)	22	
	Her2+	5 (31.25)	11 (68.75)	16	
	TN	2 (18.18)	9 (81.82)	11	
	Missing	9	13	22	
pN	Negative	25 (25.00)	75 (75.00)	100	0.004 *
	Positive	32 (46.38)	37 (53.62)	69	
	Px	6 (42.86)	8 (57.14)	14	
pT	Is	8 (38.10)	13 (61.90)	21	0.045
	1	32 (27.59)	84 (72.41)	116	
	2	14 (43.75)	18 (56.25)	32	
	3	8 (61.54)	5 (38.46)	13	
	Missing	1		1	

* Excluding Px from the test.

**Table 2:** Differentially methylated regions listing each CpG probe mapping within the three *DMRs*.

DMRindex	Chromosome	Cpg Probe ID	Position	Strand	Type	Gene	Feature	Cgi
*DMR_1*	5	cg07158503	135415693	R	II		IGR	shore
		cg04515200	135415762	F	II		IGR	shore
		cg13581155	135415781	F	II		IGR	shore
		cg11608150	135415948	R	I		IGR	shore
		cg06478886	135416029	R	II		IGR	shore
		cg04481923	135416205	R	II	VTRNA2–1	Body	island
		cg18678645	135416331	R	II	VTRNA2–1	TSS200	island
		cg06536614	135416381	F	I	VTRNA2–1	TSS200	island
		cg25340688	135416398	F	I	VTRNA2–1	TSS200	island
		cg26896946	135416405	F	I	VTRNA2–1	TSS200	island
		cg00124993	135416412	F	I	VTRNA2–1	TSS200	island
		cg08745965	135416529	F	II	VTRNA2–1	TSS1500	shore
*DMR_2*	5	cg19626725	178986131	F	II	RUFY1	TSS1500	shore
		cg00080972	178986291	F	II	RUFY1	TSS1500	shore
		cg21226059	178986404	F	II	RUFY1	TSS1500	shore
		cg14820908	178986412	F	II	RUFY1	TSS1500	shore
		cg02136620	178986620	F	II	RUFY1	TSS200	island
		cg09060608	178986726	R	II	RUFY1	TSS200	island
		cg05457628	178986728	R	I	RUFY1	TSS200	island
		cg22764044	178986830	F	II	RUFY1	1stExon	island
		cg26516362	178986906	F	I	RUFY1	5′UTR	island
*DMR_3*	10	cg06791446	123355268	F	II	FGFR2	5′UTR	shore
		cg25052156	123355454	F	II	FGFR2	5′UTR	shore
		cg22633036	123355576	R	II	FGFR2	5′UTR	shore
		cg11430259	123355748	R	I	FGFR2	5′UTR	shore
		cg02210151	123356041	R	II	FGFR2	5′UTR	shore
		cg17681491	123356205	R	II	FGFR2	5′UTR	shore
		cg18566515	123356236	R	I	FGFR2	5′UTR	shore

The table describes the CpG probe IDs and positions of the three differentially methylated regions (DMR)s. The gene and the forward or reverse designation of the designed strand are reported. Type relates to the Infinium design (Infinium I applies 2 probes/locus, while Infinium II uses 1 probe/locus). The gene region features describing the CpG region are listed below in the same order as the target gene transcripts. IGR = Intergenic region, located between genes. Body = Between the ATG and stop codon; irrespective of the presence of introns, exons, TSS (transcriptional start site), or promoters. TSS200: 0–200 bases upstream of the TSS. TSS1500: 200–1500 bases upstream of the (TSS). 5′UTR = Within the 5′ untranslated region, between the TSS and the ATG start site (start codon). island = CpG island. shore = Region within 2 kb up- or downstream of a CpG island.

**Table 3:** A summary of the biological mechanisms epigenetically regulated in our study, genes between brackets did not reach statistical significance in our study.

Mechanism	Genes Involved	Biological Impact
Cell Migration	*RUFY1*, (*CCL21*), (*CDH4*)	Immune cell recruitment, cancer invasion
Growth Signalling	*FGFR2*, (*IR*)	Proliferation, metabolic dysregulation
Adhesion/Junctions	*vtRNA2–1*, (*CDH4*)	EMT, barrier integrity
Trafficking	*RUFY1*	Receptor recycling, signalling modulation
Glucose regulation	*RUFY1*, *vtRNA2–1*, (*IR*)	Glucose regulation—Warburg effect

## Data Availability

Internal policies and ongoing projects currently prevent us from publicly sharing the data supporting this study on open platforms. However, DNA methylation profiles of de-identified participant data underlying this article may be shared after describing the proposed use of the data and obtaining approval from the IEO Data Protection Board. The request may be sent to the corresponding author or the Director of the Division of Cancer Prevention and Genetics, IEO, Milan.

## Supplementary Materials

The following supporting information can be downloaded at: https://www.mdpi.com/article/10.3390/cancers17193132/s1, Table S1: Epigenome-wide differentially methylated probes listing the first 100 single CpGs ordered by nominal *p*-value; Table S2: Odd Ratios and 95%CI from logistic regression models using the derived categorical variables, adjusting for age, array position, and estimated WBC; Table S3: Data extracted from the EPIGEN MeQTL Database; Table S4: Methylation profile scores as surrogate estimators of human traits; Figure S1: Density plots of CpG sites within DMRs.
