# Supplementary material for: Impact of Epigenome-Wide Methylation and Breast Cancer Recurrence in Women Tested Negative for BRCA Genes: The Breast Methylation Risk (BREMERI) Study
Source: Cancers (Basel). 2025 Sep 26;17(19):3132. doi: 10.3390/cancers17193132 (PMC12524240; doi:10.3390/cancers17193132)
Supplement: Supplementary file 1 [file cancers-17-03132-s001.zip › cancers-3829893-supplementary.pdf]

Table S1: Epigenome-wide differentially methylated probes listing the first 100 single CpGs ordered by nominal p-value

CpG Probe ID	Chr	Position	Gene	Feature	cgi	logFC	P-Value	deltaBeta
cg03661616	10	112327538	SMC3	5'UTR	island	0.012	0.00001	-0.012
cg05181799	3	119500059	NR1I2	1stExon	opensea	0.091	0.00001	-0.091
cg09908442	5	119663294		IGR	opensea	0.042	0.00002	-0.042
cg02646182	2	23253383		IGR	opensea	-0.037	0.00003	0.037
cg16034850	2	61071062		IGR	opensea	0.012	0.00004	-0.012
cg26691074	18	50463502	DCC	Body	opensea	-0.043	0.00005	0.043
cg15350949	5	52387628	ITGA2	3'UTR	opensea	0.018	0.00006	-0.018
cg07554011	11	537737	LRRC56	5'UTR	shore	0.025	0.00007	-0.025
cg09933454	12	3158750		IGR	opensea	-0.041	0.00007	0.041
cg25691026	19	8670586	ADAMTS10	Body	shore	0.010	0.00008	-0.010
cg08056108	19	34636510		IGR	opensea	-0.039	0.00008	0.039
cg09402652	19	3961645	MIR637	TSS200	shore	-0.013	0.00009	0.013
cg23213983	16	57224974	RSPRY1	5'UTR	opensea	0.026	0.00010	-0.026
cg13832333	16	87329470		IGR	opensea	0.065	0.00012	-0.065
cg02430368	6	25651724	SCGN	TSS1500	shore	-0.051	0.00013	0.051
cg23828422	12	76308784		IGR	opensea	0.015	0.00015	-0.015
cg25012529	13	113793489	F10	Body	opensea	0.040	0.00018	-0.040
cg20577102	20	39966297		IGR	shelf	0.025	0.00018	-0.025
cg23791325	2	95743038		IGR	shore	0.028	0.00018	-0.028
cg21373546	19	50191788	ADM5	TSS200	shore	-0.017	0.00018	0.017
cg24063036	17	76464748	DNAH17	Body	opensea	-0.015	0.00019	0.015
cg13038078	3	2280346	CNTN4	TSS200	opensea	0.023	0.00019	-0.023
cg13658348	21	37762251	CHAF1B	Body	opensea	-0.008	0.00019	0.008
cg11882845	12	58063680		IGR	opensea	-0.012	0.00020	0.012
cg23980760	11	44069581	ACCSL	1stExon	opensea	-0.012	0.00022	0.012
cg21354037	3	188580381	LPP	Body	opensea	-0.010	0.00024	0.010
cg04411342	3	133103339	TMEM108	Body	opensea	-0.037	0.00024	0.037
cg07323991	8	108326384	ANGPT1	Body	opensea	0.025	0.00025	-0.025
cg06447614	16	1195491		IGR	shelf	-0.023	0.00025	0.023
cg08723318	10	122723063		IGR	opensea	-0.035	0.00025	0.035
cg03641585	17	10761400		IGR	opensea	-0.086	0.00026	0.086
cg01810111	6	27637395		IGR	opensea	-0.017	0.00027	0.017

cg14620100	13	97406733	HS6ST3	Body	opensea	0.015	0.00029	-0.015
cg19206728	12	39734162	KIF21A	ExonBnd	opensea	0.024	0.00029	-0.024
cg17582411	17	77458542	HRNBP3	5'UTR	shore	0.016	0.00030	-0.016
cg11269103	11	6976223	ZNF215	Body	opensea	-0.021	0.00030	0.021
cg03561212	16	12733143		IGR	opensea	-0.047	0.00031	0.047
cg11371118	6	160683689		IGR	shelf	-0.038	0.00031	0.038
cg02615938	16	24635788		IGR	opensea	-0.030	0.00032	0.030
cg18561976	2	204801508	ICOS	5'UTR	opensea	-0.044	0.00033	0.044
cg09322322	17	77288385	RBFOX3	5'UTR	opensea	-0.029	0.00033	0.029
cg17240112	16	77694375		IGR	opensea	-0.052	0.00034	0.052
cg00694096	4	31413716		IGR	opensea	0.029	0.00034	-0.029
cg07269003	3	142681516	PAQR9	1stExon	island	-0.018	0.00036	0.018
cg14391245	14	94840176		IGR	opensea	-0.018	0.00038	0.018
cg01802114	10	62204885	ANK3	Body	opensea	0.025	0.00039	-0.025
cg00501035	4	22754557	GBA3	Body	opensea	0.031	0.00040	-0.031
cg12224203	17	10761460		IGR	opensea	-0.050	0.00040	0.050
cg05723130	10	124248994	HTRA1	Body	opensea	-0.006	0.00042	0.006
cg20487890	1	185402344		IGR	opensea	-0.012	0.00043	0.012
cg05785424	17	21226409		IGR	opensea	-0.063	0.00043	0.063
cg16397021	15	25953388	ATP10A	Body	opensea	0.007	0.00043	-0.007
cg03199903	14	71122637	TTC9	Body	opensea	0.045	0.00044	-0.045
cg27056759	14	105373391		IGR	shore	-0.041	0.00044	0.041
cg13556012	17	29808380	RAB11FIP4	Body	opensea	-0.006	0.00045	0.006
cg03095758	1	235018931		IGR	opensea	0.029	0.00045	-0.029
cg07124045	10	103595509	KCNIP2	Body	opensea	0.020	0.00046	-0.020
cg09279930	8	43232712		IGR	opensea	-0.043	0.00046	0.043
cg07402003	16	52503883	TOX3	Body	opensea	0.014	0.00046	-0.014
cg21402640	3	126135211	CCDC37	Body	island	-0.026	0.00046	0.026
cg16422492	16	31228555	TRIM72	Body	shore	-0.018	0.00047	0.018
cg13636978	8	15598297	TUSC3	Body	opensea	0.015	0.00048	-0.015
cg18414021	14	100069535	CCDC85C	1stExon	island	0.024	0.00048	-0.024
cg10719522	13	28562841	PRHOXNB	TSS200	opensea	-0.010	0.00049	0.010
cg15552461	19	55581114	RDH13	TSS1500	opensea	-0.031	0.00050	0.031
cg21909219	18	72415107	ZNF407	Body	opensea	-0.011	0.00051	0.011

cg09670971	19	888612	MED16	Body	shelf	0.092	0.00051	-0.092
cg16769260	10	78084390	C10orf11	Body	opensea	0.015	0.00052	-0.015
cg22838050	19	872690	MED16	Body	shore	0.064	0.00053	-0.064
cg00328434	11	9094580	SCUBE2	Body	opensea	-0.023	0.00054	0.023
cg10856266	10	130165309		IGR	opensea	0.013	0.00055	-0.013
cg27535320	16	82637682		IGR	opensea	-0.015	0.00056	0.015
cg07911664	13	112627528		IGR	island	-0.043	0.00056	0.043
cg25761371	19	6712479	C3	Body	shore	-0.009	0.00056	0.009
cg24052851	9	90795355		IGR	island	-0.005	0.00056	0.005
cg08906246	4	5167599	STK32B	Body	opensea	-0.036	0.00056	0.036
cg00310100	3	9597046		IGR	shore	-0.028	0.00058	0.028
cg08256536	8	124215129	FAM83A	Body	opensea	-0.021	0.00060	0.021
cg14356550	1	18808102	KLHDC7A	1stExon	shore	-0.026	0.00060	0.026
cg14778875	8	70461961	SULF1	5'UTR	opensea	-0.014	0.00061	0.014
cg07551380	18	10560797		IGR	opensea	-0.035	0.00062	0.035
cg02739364	21	43987405	SLC37A1	Body	shelf	-0.005	0.00064	0.005
cg10934718	2	39667058	LOC728730	Body	shelf	-0.015	0.00066	0.015
cg13399071	12	121431394	HNF1A	Body	opensea	-0.013	0.00066	0.013
cg04072850	8	24241186	ADAMDEC1	TSS1500	opensea	0.032	0.00066	-0.032
cg13433125	17	67957680	LINC01497	TSS200	opensea	-0.023	0.00067	0.023
cg24473523	5	148758982	IL17B	TSS200	opensea	-0.009	0.00067	0.009
cg17724175	1	150552817	MCL1	TSS1500	shore	-0.045	0.00068	0.045
cg11148296	12	95645155	VEZT	Body	opensea	0.025	0.00068	-0.025
cg14756480	18	39534432	PIK3C3	TSS1500	opensea	0.015	0.00069	-0.015
cg21950155	13	28555387	PRHOXNB	Body	shore	-0.088	0.00069	0.088
cg09488594	5	60602794		IGR	opensea	-0.020	0.00069	0.020
cg15345477	10	130279644		IGR	opensea	-0.043	0.00069	0.043
cg08728118	6	14965804		IGR	opensea	-0.005	0.00070	0.005
cg12181907	12	118681284	TAOK3	Body	opensea	0.022	0.00071	-0.022
cg05470393	2	204801413	ICOS	TSS200	opensea	-0.038	0.00071	0.038
cg15765251	19	888814	MED16	Body	shelf	0.061	0.00072	-0.061
cg14549869	9	37577490	FBXO10	TSS1500	shore	-0.005	0.00072	0.005
cg05768565	4	7553433	SORCS2	Body	opensea	-0.008	0.00072	0.008
cg10030980	7	81792131	CACNA2D1	Body	opensea	0.037	0.00073	-0.037

Features are defined as follows: TSS200 = 0–200 bases upstream of the transcriptional start site (TSS); TSS1500 = 200–1500 bases upstream of the TSS; 5'UTR = Within the 5' untranslated region, between the TSS and the ATG start site; Body = Between the ATG and stop codon; irrespective of the presence of introns, exons, TSS, or promoters; IGR = intergenic region; 3'UTR = Between the stop codon and poly A signal

Table S2: Odd Ratios and 95%CI from logistic regression models using the derived categorical variables, adjusting for age, array position, and estimated WBC

		OR	Low 95%CI	Up 95%CI	p-value
DMR1	cg07158503	0.79	0.37	1.66	0.53
	cg04515200	0.72	0.35	1.52	0.39
	cg13581155	0.83	0.40	1.71	0.62
	cg11608150	0.73	0.36	1.48	0.39
	cg06478886	0.79	0.37	1.66	0.53
	cg04481923	0.66	0.32	1.33	0.24
	cg18678645	0.77	0.38	1.59	0.48
	cg06536614	0.76	0.37	1.54	0.44
	cg25340688	0.73	0.36	1.47	0.38
	cg26896946	0.68	0.34	1.35	0.27
	cg00124993	0.77	0.38	1.55	0.46
	cg08745965	0.77	0.38	1.59	0.48
	cg18797653	0.73	0.36	1.47	0.38
DMR3	cg06791446	0.80	0.39	1.62	0.53
	cg25052156	0.88	0.43	1.78	0.72
	cg22633036	0.57	0.29	1.13	0.11
	cg02210151	0.82	0.41	1.67	0.59
	cg18566515	0.61	0.30	1.24	0.17

Table S3: Data extracted from the EPIGEN MeQTL Database (https://epicmeqtl.kcl.ac.uk/#tab-8866-2 accessed on 17 September 2025)

CpG Probe ID	DMR	meQTL SNP ID	Chr	CpG position	SNP position	Effect Allele	MAF	Beta	SE	P	FDR	Cis/Trans	Note
cg07158503	DMR1	5:135415726_G_A	5	135415693	135415726	A	0.354	-0,22	0,0351	5,28E-10	1,44E-06	cis	
cg04515200	DMR1	5:135440176_T_C	5	135415762	135440176	C	0.166	-0,28	0,0493	9,08E-09	1,55E-05	cis	
cg13581155	DMR1												No meQTLs
cg11608150	DMR1	5:135402852_C_G	5	135415948	135402852	G	0.315	-0,23	0,0383	2,09E-09	4,48E-06	cis	
cg06478886	DMR1	5:135403851_A_G	5	135416029	135403851	G	0.207	-0,32	0,0417	2,51E-14	0	cis	
cg04481923	DMR1												No meQTLs
cg18678645	DMR1	5:135414280_G_A	5	135416331	135414280	A	0.353	-0,20	0,0509	0,000127	0,0328	cis	
cg06536614	DMR1												No meQTLs
cg25340688	DMR1												No meQTLs
cg26896946	DMR1	5:134606269_T_G	5	135416405	134606269	G	0.203	0,27	0,0708	0,000176	0,0421	cis	
cg00124993	DMR1												No meQTLs
cg08745965	DMR1												No meQTLs
cg18797653	DMR1												No meQTLs
cg06791446	DMR3	10:123175486_T_A	10	123355268	123175486	A	0.459	-0,86	0,0317	4,39E-162	0	cis	
cg25052156	DMR3	10:123175486_T_A	10	123355454	123175486	A	0.459	-0,86	0,0316	2,39E-163	0	cis	
cg22633036	DMR3	10:123175486_T_A	10	123355576	123175486	A	0.459	-0,85	0,0316	9,01E-158	0	cis	
cg11430259	DMR3	10:123228500_A_G	10	123355748	123228500	G	0.236	0,84	0,0335	1,11E-137	0	cis	
cg02210151	DMR3	10:123175486_T_A	10	123356041	123175486	A	0.459	-0,87	0,0314	5,31E-171	0	cis	
cg17681491	DMR3	10:123175486_T_A	10	123356205	123175486	A	0.459	-0,82	0,0323	1,83E-140	0	cis	
cg18566515	DMR3	10:123175486_T_A	10	123356236	123175486	A	0.459	-0,79	0,0318	1,27E-135	0	cis	

meQTL SNP ID: SNP ID in the format chr:SNPposition _OtherAllele_ Effect Allele; Chr: chromosome for the SNPs and CpGs; CpG and SNP positions: position of CpG and SNP on the chromosome; Effect Allele: allele associated with the change in methylation at the CpG site; MAF: minor allele frequency for the SNP; Beta: association coefficient estimate, with respect to the effect allele; SE: standard error of β coefficient; P: nominal P-value of the β coefficient; FDR: P-value after multiple testing adjustment with the permutation approach; Cis/Trans: type of association, can be cis (< 1 Mbp between top SNP and CpG) or trans (all others).

Table S4: Methylation profile scores as surrogate estimators of human traits

Metabolic traits	Estimate	Standard Error	P-value
CCL21	89.06	40.52	0.028
Insulin receptor (INSR)	94.14	46	0.041
NTRK3	-55.48	28.94	0.055
Neutral ceramidase	-66.72	36.28	0.066
CXCL11	42.21	24.41	0.084
RARRES2	59.91	34.75	0.085
CRP	61.11	36.85	0.097
ENPP7	-12.51	7.55	0.098
Testican 2	87.52	56.09	0.119
FCER2	43.76	28.91	0.130

Results of the linear models in which each trait score was used as the outcome (dependent variable) and the status of case or control as the predictor, adjusting for age, WBCs estimated percentage, and chipposition (SentrixID + SentrixPosition). The table is limited to the first 10 traits. P-values are adjusted for false discovery rate (FDR).

Figure S1

a) Density plots of CpG sites. DMR1: VTRNA1-2

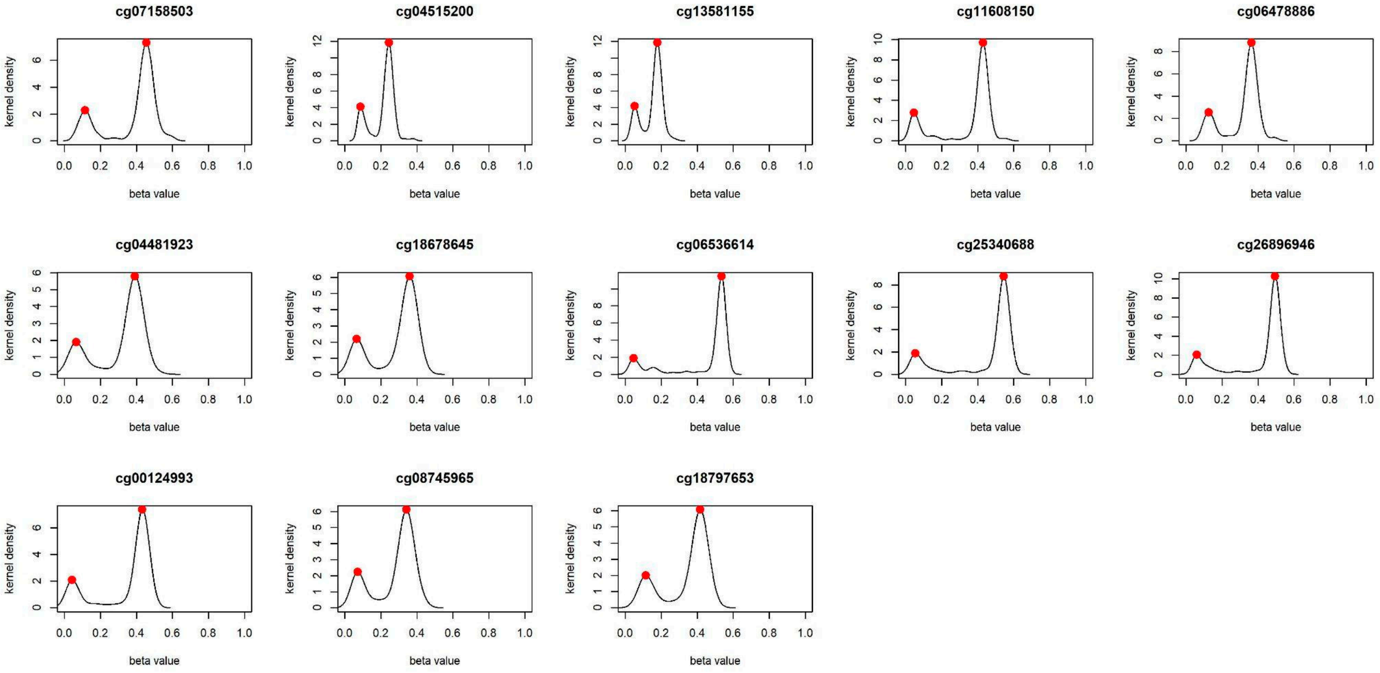

b) Density plots of CpG sites DMR2: RUFY1

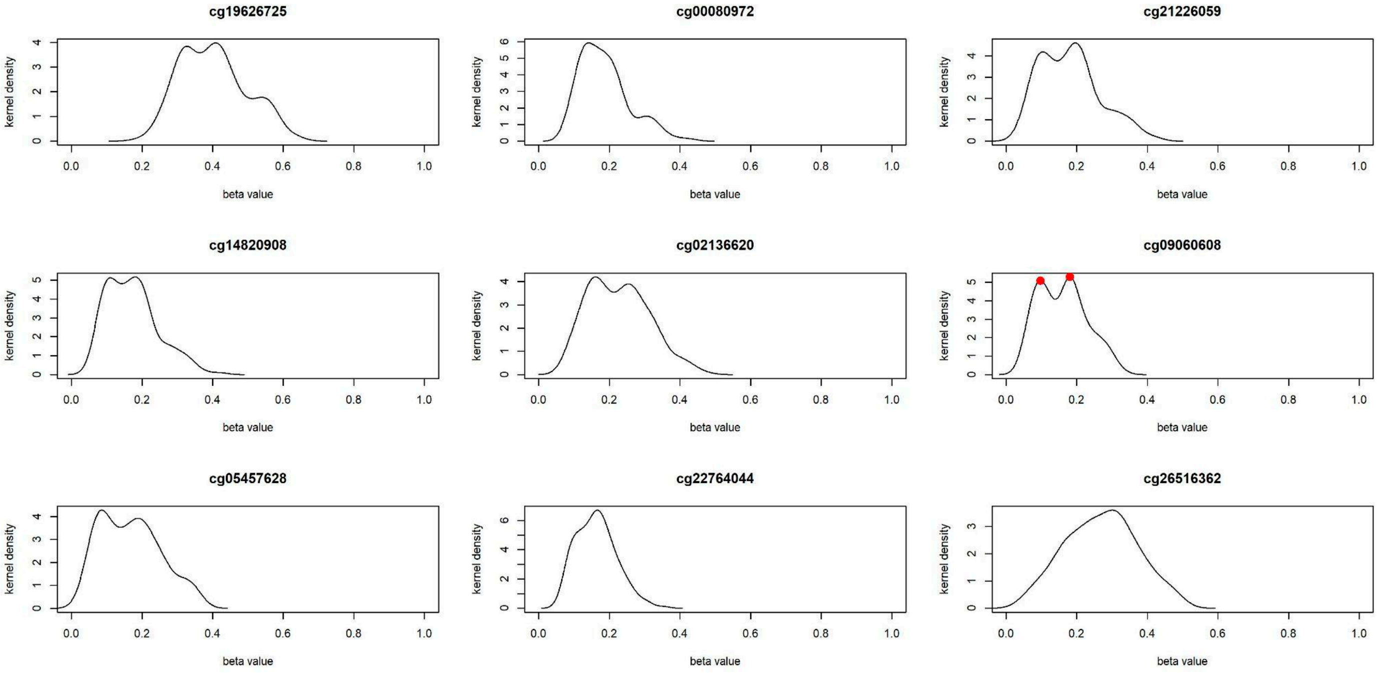

c) Density plots of CpG sites DMR3: FGFR2

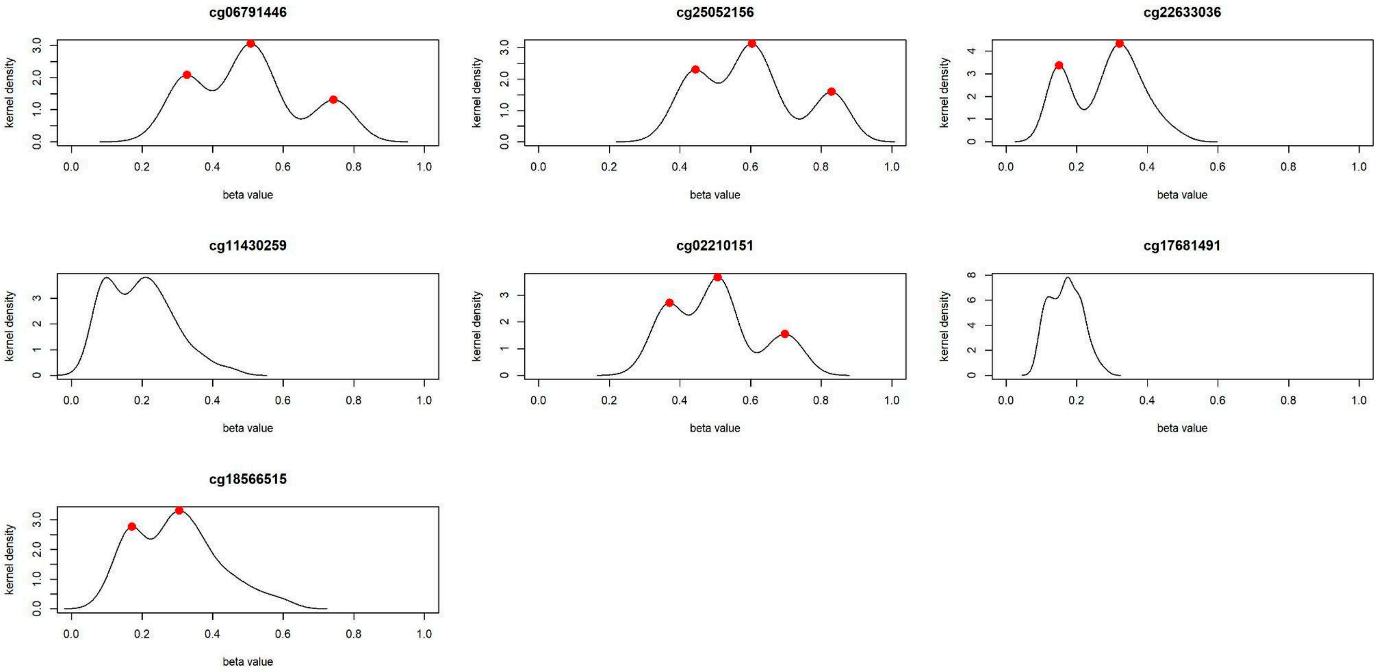

Legends

Density plots of CpG sites within DMRs. For each CpG site, methylation β-values are shown on the x-axis and the corresponding kernel density estimates on the y-axis. CpGs exhibiting evidence of multimodality, as determined by Hartigan's dip test, are highlighted with red dots marking the density peaks. (a) DMR1: VTRNA1-2; (b) DMR2: RUFY1; (c) DMR3: FGFR2
